# Fellow-Workers and the Roll of Honour

**Published:** 1915-04-24

**Authors:** 


### ROUND THE WORLD.

[The Editor invites Early Information and Contributions Marked "Fellow-Workers."]

### Fellow-Workers and the Roll of Honour.

The country will have good cause to be thankful for the keen work of those medical men who have during recent years developed the comparatively new science of hygiene to its present pitch of practical efficiency. Many medical officers of health and assistants have accepted commissions in the various sanitary corps of the new Armies, and in addition specialists in this branch of medicine are being appointed to particular duties and investigations under the ægis of the War Office. Such an appointment is that of Dr. A. Middleton Hewat.

An M.D. of Edinburgh University and a D.P.H. of Edinburgh and Glasgow, Dr. Aubrey Middleton Hewat is assistant medical officer of health for the county borough of Preston, as well as tuberculosis officer for the same district. The special work now placed in his hands is of the utmost importance, for as bacteriologist to the Western Command he is to pursue an inquiry into the occurrence of cerebro-spinal meningitis amongst the troops in Lancashire, Cumberland, and Westmorland.

Mr. Archer Ryland, F.R.C.S. Edin., who has taken a commission as lieutenant in the R.A.M.C., and been appointed otologist to the Military Hospital at Frensham Hill, has lately been working at the Central London Throat and Ear Hospital as surgical registrar. After qualifying at St. Bartholomew's he served as house surgeon there, as well as at the Great Northern Central Hospital and at the Throat Hospital just mentioned.

During Dr. Lennane's absence the duties of medical officer of health for Battersea are being carried out by Dr. Charles Edmund McDade, who is well known in the locality, where he has for some time been public vaccinator and medical officer to No. 3 district. A Belfast and King's College student, he took the M.D. of the Royal University of Ireland and the D.P.H. of the Royal College of Surgeons. At Belfast he won the Malcolm Exhibition of the Royal Hospital there.

At the Royal Eye Hospital, Southwark, three appointments have just been made as temporary assistant surgeons, the gentlemen selected for this work being Mr. F. D. Bennett, Mr. T. W. Letchworth, and Mr. A. E. A. Loosely.

Mr. Francis Dillon Bennett, L.R.C.P., M.R.C.S., L.S.A., is a senior clinical assistant at the Royal Eye Hospital, and was formerly anæsthetist to the Royal Ear Hospital, Soho. He is medical officer to the Army and Navy Stores and to the Legal and General Assurance Company. After leaving "Bart.'s," where he received his medical education, Mr. Bennett held the posts of resident surgeon to the Manchester Ship Canal Hospital and house surgeon to the East Suffolk Hospital.

Mr. Thomas Wilfrid Letchworth, M.B. Cantab, F.R.C.S. Eng., is also a "Bart.'s" man who has specialised in ophthalmic surgery. Formerly house surgeon to the Royal Westminster Ophthalmic Hospital, his present appointments include those of ophthalmic surgeon to the Western General Dispensary, the Hospital of St. John and St. Elizabeth, and to the Tottenham and Surrey Education Authorities.

Mr. Alfred Edward Arthur Loosely, M.B. Oxon, F.R.C.S. Eng., went to St. Thomas's Hospital on leaving Oxford, and after qualifying held the posts of senior and junior ophthalmic house surgeon at that institution. He is now a chief clinical assistant at the Royal London Ophthalmic Hospital and ophthalmic surgeon to the Hounslow Hospital. At the Royal Eye Hospital he has been working as assistant for refraction.

Interest necessarily is maintained in the important problem of frost-bite amongst the troops at the Front, and it is to be hoped that recent experiences will enable trustworthy preventive measures to be taken should our men have to face the rigours of another winter campaign. Amongst the latest contributions to the literature of the subject must be mentioned reports communicated to the *Lancet* by Major Miller, Mr. Loughnane, and Captain Max Page. A French view was given in our Notes last week.

Major Charles Hewitt Miller, after analysing nearly 400 cases of frost-bite observed at the British Red Cross Hospital, Netley, concludes that the condition is primarily physical. Major Miller, who now holds a commission (temporary) in the R.A.M.C., is in peace time assistant physician to the London Hospital, where he had a distinguished career as a student, holding various junior posts after qualification, and having for a time the care of the special Tyrnauer hot-air baths at that institution. Taking the diploma of the Conjoint Board in 1901, he subsequently became an M.D. Cantab., M.R.C.P. Lond.

Mr. Farquhar McGillivray Loughnane is resident surgical officer to Salford Royal Hospital, but has lately been working with the Army as British Red Cross surgeon at the Meerut Stationary Hospital, Boulogne. His report is particularly interesting as dealing with the incidence of frost-bite amongst the Indian soldiers, and it is to be noted that, contrary to what might have been expected, he is of opinion that they stood the winter as well as the Europeans.

Captain Charles Max Page, who is an officer of the R.A.M.C. Special Reserve, has reported on observations made in one of the large base hospitals on the Continent, where several hundred cases of frost-bite came under notice. He appears to be of opinion that there is a nervous factor in some of the cases. A distinguished student of St. Thomas's Hospital, where he holds the post of demonstrator of anatomy and was formerly resident assistant surgeon and surgical registrar, Captain Max Page took the M.B., B.S. Lond. with honours, following up this success by winning the gold medal at the M.S. Lond., and becoming an F.R.C.S. Eng. He is on the staff of the Victoria Hospital for Children, being one of the out-patient surgeons there. During the Balkan Wars he served under the British Red Cross with the Turkish Detachment.

Major John Henry Harris, R.A.M.C.(T.), who has been appointed specialist in surgery to the 4th Wessex Brigade R.F.A., is senior surgeon to the Dartmouth Hospital, medical officer of health for the Borough of Dartmouth, and medical inspector of seamen there. An M.D. Durh., M.R.C.S. Eng., D.P.H. Cantab., he qualified L.S.A. from St. Bartholomew's in 1883. In addition to the duties entailed by the post mentioned, he has done a good deal of work for the Post Office and various insurance companies. Major Harris's new appointment is connected with the temporary station of his unit in India.

